# Whole-Genome Sequences of *Brucella melitensis* Strain QH61, Isolated from Yak in Qinghai, China

**DOI:** 10.1128/genomeA.01422-17

**Published:** 2018-01-11

**Authors:** Xiaoan Cao, Zhaocai Li, Zhongzi Lou, Baoquan Fu, Yongsheng Liu, Youjun Shang, Jizhang Zhou, Zhizhong Jing

**Affiliations:** a State Key Laboratory of Veterinary Etiological Biology, Lanzhou Veterinary Research Institute, Chinese Academy of Agricultural Sciences, Lanzhou, Gansu, People’s Republic of China

## Abstract

The facultative intracellular Gram-negative bacterium *Brucella melitensis* causes brucellosis in domestic and wild mammals. *Brucella melitensis* QH61 was isolated from a yak suffering from abortion in 2015 in Qinghai, China. Here, we report the whole-genome sequence of *B. melitensis* strain QH61.

## GENOME ANNOUNCEMENT

*Brucella* spp. are Gram-negative and facultative intracelluar pathogens that cause animal brucellosis, a disease that leads to abortion and infertility in livestock and severe economic losses and threats to health in humans (1, 2). In recent years, brucellosis has reemerged in animals in China. *Brucella melitensis* not only causes the most cases of brucellosis in animals but also is an important pathogen responsible for human disease (3, 4).

Here, we present the genome sequence of *B. melitensis* QH61, which was isolated in China from the spleen of an aborted yak fetus. This is the first report of the whole-genome sequencing of a *B. melitensis* strain isolated from yak in China.

Genomic DNA was extracted with the DNeasy blood and tissue kit (Qiagen, Valencia, CA). This genome was sequenced by using PacBio technology after library construction with PacBio RS II and Illumina HiSeq DNA to determine the complete genomic sequence of the *B. melitensis* QH61. The annotation was performed using GeneMarkS (5), Repeat Masker (6), TRF (7), tRNAscan-SE (8), and RNAmmer (9).

The whole-genome sequence of *B. melitensis* QH61 was found to be 3,311,767 bp in size and to be composed of two circular chromosomes, chromosome I (2,126,173 bp) and chromosome II (1,185,594 bp). The G+C content of this strain is 58.34%. Approximately 86.67% (2,870,451 bp) of the nucleotide sequences were predicted to be coding sequences containing 3,317 genes, and the average gene length was 865 bp. This strain has 2 small RNAs (sRNAs), 55 tRNAs, and 3 rRNAs (1 23S, 1 16S, and 1 5S). A total of 2,258 (68.1%) of 3,317 protein-coding genes had at least some biological function assigned, with some of the genes assigned to more than one category (10).

### Accession number(s).

The whole-genome sequence of *B. melitensis* QH61 has been deposited at DDBJ/EMBL/GenBank under the accession no. CP024653 and CP024654.
